# Associations Between Supported Accommodation and Health and Re-offending Outcomes: a Retrospective Data Linkage Study

**DOI:** 10.1007/s11524-023-00824-w

**Published:** 2024-02-13

**Authors:** Daisy Gibbs, Samantha Colledge-Frisby, Sara Farnbach, Michael Doyle, Anthony Shakeshaft, Sarah Larney

**Affiliations:** 1 National Drug and Alcohol Research Centre, UNSW Sydney, 22-32 King Street, Randwick, NSW 2031 Australia; 2 National Drug Research Institute, Curtin University, Perth, Australia; 3 Central Clinical School, University of Sydney, Sydney, Australia; 4 Poche Centre for Urban Indigenous Health, University of Queensland, Brisbane, Queensland Australia; 5 Department of Family Medicine and Emergency Medicine, Universite de Montreal and Centre de Recherche du CHUM, Montreal, Canada

**Keywords:** Supported accommodation, Prison, Reincarceration, Post-release accommodation

## Abstract

Following release from prison, housing and health issues form a complex and mutually reinforcing dynamic, increasing reincarceration risk. Supported accommodation aims to mitigate these post-release challenges. We describe the impact of attending Rainbow Lodge (RL), a post-release supported accommodation service for men in Sydney, Australia, on criminal justice and emergency health outcomes. Our retrospective cohort study using linked administrative data includes 415 individuals referred to RL between January 2015 and October 2020. Outcomes of interest were rates of criminal charges, emergency department (ED) presentations and ambulance attendance; and time to first reincarceration, criminal charge, ED presentation and ambulance attendance. The exposure of interest was attending RL; covariates included demographic characteristics, release year and prior criminal justice and emergency health contact. Those who attended RL (*n* = 170, 41%) more commonly identified as Aboriginal or Torres Strait Islander (52% vs 41%; *p* = 0.025). There was strong evidence that attending RL reduced the incidence criminal charges (adjusted rate ratio [ARR] = 0.56; 95% confidence interval [CI] 0.340.86; *p* = 0.009). Absolute rates indicate a weak protective effect of RL attendance on ED presentation and ambulance attendance; however, adjusted analyses indicated no evidence of an association between attending RL and rates of ED presentations (ARR = 0.88; 95% CI = 0.65–1.21), or ambulance attendance (ARR = 0.82; 95% CI = 0.57–1.18). There was no evidence of an association between attending RL and time to first reincarceration, charge, ED presentation or ambulance attendance. Greater detail about reasons for emergency health service contact and other self-report outcome measures may better inform how supported accommodation is meeting its intended aims.

**Supplementary Information:**

The online version contains supplementary material available at 10.1007/s11524-023-00824-w.

## Introduction

In 2021, half of people released from prison in Australia (54%) expected to be homeless [1]. The relationship between housing, health and incarceration is complex and often mutually reinforcing [2–6]. Unstable housing following release from prison is associated with an increased risk of reincarceration [7]. Amongst newly incarcerated individuals, those who reported being homeless prior to incarceration were are also more likely to be reincarcerated that those who had been housed [8], demonstrating further the unique dynamic between incarceration and homelessness [5, 9]. Supported accommodation aims to provide a secure environment that mitigates the housing, health and incarceration issues typically encountered in the vulnerable period following release from prison.

Supported accommodation may take different forms, including group homes or scattered site housing, coupled with case management or other therapeutic activities [10]. Despite the role supported accommodation may play for people released from prison, a 2023 systematic review found only seven evaluations of these services [10], five of which were conducted in the USA [11–15]. In these evaluations, the lack of standardisation across both service delivery and evaluation design was evident, with 15 different outcome measures used to demonstrate the impact of supported accommodation. This dearth of consistent evidence leaves service providers and policymakers poorly equipped to decide how to design and deliver supported accommodation for people released from prison. This heterogeneity also reduces opportunities for researchers to pool samples from evaluations in meta-analyses, which would increase statistical power and might improve the usefulness of the existing research.

A co-designed model of care and program logic could clarify how supported accommodation program components are expected to achieve their defined outcomes and more directly align the program’s aims with standardised outcome measures to assess effectiveness [16–18]. Our research team recently proposed a model of care and program logic that was co-designed by integrating the best-available research evidence with the expertise of staff and the lived experience of clients of The Rainbow Lodge Program (RL), a supported accommodation service for men released from prison in Sydney, Australia. Reduced incidence of criminal charges and longer time to reincarceration were two of the primary outcomes included in the program logic, with other outcomes related to quality of life and self-efficacy [19].

The use of routinely collected administrative data to assess health and criminal justice outcomes has the advantage of increasing the size of the population of interest, being relatively inexpensive and being less likely to be biased by non-consent or recall bias than self-report data [20]. Linked administrative data enables a low-resource means of measuring the impact of attending supported accommodation on criminal justice and health outcomes included in the co-designed model of care and program logic. Emergency department (ED) presentation after release from prison is commonly due to health problems that are also associated with reincarceration, primarily alcohol and other drug use or mental health issues [21–23], and frequent contact with emergency health services (ED and ambulance attendance) is itself associated with increased likelihood of reincarceration [24]. Although self-efficacy and quality of life may be better measured using self-report data, health service utilisation may provide proxy or complementary data to inform assessments of service effectiveness.

We conducted a retrospective cohort study using state-wide linked administrative health and criminal justice data in New South Wales (NSW), Australia, to achieve the following aims:

i. determine the feasibility of using linked administrative data to assess the impact of attending supported accommodation on health and criminal justice outcomes following release from prison;
ii. compare rates of health and re-offending outcomes (ED presentation, ambulance attendance, criminal charges) between people who did and did not attend RL; and
iii. determine if program participation is associated with a longer time to first health service or criminal justice system contact (ED presentation, ambulance attendance, criminal charge, reincarceration).

## Methods

A retrospective cohort study using linked, state-wide, administrative health and criminal justice data in NSW, Australia.

This paper is reported in line with the Reporting of studies Conducted using Observational Routinely-collected health Data Statement (RECORD; Appendix A) [25]. Approval for this study was obtained from the NSW Population and Health Services Research Ethics Committee (2020.82), the University of New South Wales Human Research Ethics Committee (HC200276), the Aboriginal Health and Medical Research Council of NSW’s Human Research Ethics Committee (1683/20) and Corrective Services NSW.

### Setting and Participants

The base cohort included everyone referred to RL between January 1 2015 and September 31 2020, captured in The Partnership and Community Engagement dataset. RL is a supported accommodation service for men released from adult custody in NSW, Australia. The service is based in metropolitan Sydney and provides 12 weeks of residential support to men who are assessed as having a medium-high to high risk of re-offending according to the level of service inventory-revised (LSI-R); have at least 12 weeks of parole and have not been charged with an offence against children or a sexual offence. The program has a capacity of eight men (reduced to seven in 2020 due to COVID-19 restrictions) and provides an additional 2 years of outreach support following the residential program.

### Data Sources and Linkage

#### The Partnership and Community Engagement (PACE) Database

The NSW Department of Communities and Justice maintains the Partnership and Community Engagement (PACE) database. PACE records all individuals referred to RL, including the date of referral, case confirmation (attended or did not attend), date of case confirmation and reason for non-attendance (summarised in Appendix B). Data on program completion (i.e., whether an individual stayed for the complete 12 weeks) or duration of stay were not available. We extracted data for referrals between January 1 2015 and September 31 2020 were included.

#### Emergency Data: NSW Emergency Department Data Collection

The NSW Ministry of Health maintains the Emergency Department Data Collection (EDDC), which records information on presentations to public EDs in NSW. Variables provided include demographics, date of ED presentation and triage category. ED contacts between January 1 2010 and December 31 2020 were included.

#### Ambulance Data: NSW Ambulance Data Collections

NSW Ambulance Data Collections capture information for emergency and urgent episodes of care for NSW Ambulance patients who were transported to a hospital, left at a scene following clinician assessment or who died at the scene. Variables provided included demographics and date of ambulance attendance. Ambulance attendances between January 1 2010 and December 31 2020 were included.

#### Death Data: NSW Registry of Births, Deaths and Marriages, NSW Cause of Death Unit Record File

The Registrar of the NSW Registry of Births, Deaths and Marriages and the NSW Cause of Death Unit Record File provided date and cause of death information. Deaths between January 1 2010 and December 31 2020 were included.

#### Re-offending and Custody Data: NSW Bureau of Crime Statistics and Reporting Re-offending Database

The NSW Bureau of Crime Statistics and Reporting (BOCSAR) Reoffending Database (ROD) contains information on each person who has been convicted of a criminal offence in NSW, including finalised Local, District, Supreme and Children’s Criminal Court actions, as well as adult and juvenile custody data. Variables include charge finalisation date, prison entry date, discharge date and demographic information. In these datasets, the reason for incarceration is not specified. The incarceration may be the result of a new charge, a technical violation of parole conditions (which would not result in a charge), or a charge predating release from index incarceration. Charges and incarceration between January 1 2010 and December 31 2020 were included.

#### Linkage

Databases were linked to individuals identified in the PACE dataset by the Centre for Health Record Linkage (CheReL) using probabilistic linkage and ChoiceMaker software [26]. Records were matched on individuals’ names, gender, date of birth and state of residence. A flowchart documenting the data-cleaning process can be found in Appendix C.

Using best practice privacy-preserving record linkage procedures, the CheReL does not use any health information in the linkage process. Only de-identified data were released to the researchers.

### Variables

#### Exposure Variable

##### RL Attendance

Participants were stratified into two groups for analysis: attending RL (referred and accepted) or not attending RL (referred and rejected). The date of entry to RL was not available; however, it was estimated based on the date of referral, date of discharge from index incarceration (i.e. incarceration which preceded referral) and date of decision about RL attendance. Participants entered the dataset at the time of release from index incarceration, with the start of follow-up defined as the latest of either release date or date of decision regarding RL attendance. Standard program attendance was 3 months from reception.

#### Outcome Variables

There were four outcomes to respond to aims two and three. ED presentations were coded as urgent if the triage category assigned to the event was resuscitation, emergency or urgent, and coded as low acuity if categorised as semi-urgent or non-urgent [27]. Ambulance attendance events include patients transported to a hospital or remaining at a scene following clinical assessments. A new criminal charge was defined as a new finalised criminal charge, determined by a new charge date during the follow-up period. Reincarceration was coded as the first day of an individual’s prison sentence.

#### Additional Variables

Age at the time of cohort entry was derived from information provided in the ROD dataset and dichotomised into < 40 and ≥ 40 years. Participants who identified as Aboriginal or Torres Strait Islander in any dataset were considered Aboriginal or Torres Strait Islander in the analyses [28]. The calendar year of release from index incarceration was categorised into three groups (2015–2016, 2017–2018 and 2019–2020). We used a look-back period of 5 years before release from index incarceration to determine recent prison sentences, criminal charges, ED presentations and ambulance attendances.

## Data Analysis

All analyses were conducted using SAS V9.4 [29]. All hypothesis tests were considered significant at *p* < 0.05.

We used descriptive statistics to examine the cohort’s demographic characteristics, health and criminal justice histories. Those who did and did not attend RL were compared using *χ*^2^ tests for categorical variables and the Wilcoxon rank-sum test for continuous variables.

Detail about the curation of the dataset and establishment of the start date and follow-up period can be found in Appendix B. Person-time started the day of release from the index incarceration. All participants had a minimum of three months (consistent with RL standard program duration) and a maximum of two years follow-up. The last date of available data across outcome datasets was December 31 2020. Therefore, the end of follow-up for each participant was the earliest of 2 years from start date, reincarceration, death or December 31 2020.

In addressing aim 2, we calculated rates per 100 person-years (PY) of ED presentations, ambulance attendance and new criminal charges following release, stratified by attendee status. The total number of outcome events was divided by total person-time overall and in each attendance group. Crude rate ratios were calculated by dividing the rate of an outcome among those who attended RL by the corresponding rate among those who did not. Poisson regression with a log link estimated the adjusted incidence rate ratio (ARR) per 100 PY with 95% confidence intervals (CIs). We adjusted for baseline characteristics that differed significantly between the two groups. We also adjusted for covariates that were significant at *p* < 0.05 in unadjusted models. We used deviance statistics to account for overdispersion in the dataset and included an offset for the log of person-time for each individual to account for differential follow-up time between individuals.

To address aim 3, we used Kaplan-Meier plots to examine trends in time from index incarceration to the first of each outcome event: ED presentation, ambulance attendance, new criminal charge and reincarceration. Cox regression survival analysis was then conducted to determine the effect of RL attendance on time-to-outcome events. If an outcome event did not occur during the participant’s follow-up period, the individual was right-censored and their follow-up days were entered as the censored time interval. Reincarceration was a censoring event in analyses of ED presentation, ambulance attendance and criminal charges, because a return to prison and subsequent release was considered a new community entry with a potentially changed risk of experiencing other outcomes. The covariate adjustment strategy was as for aim 2.

## Results

### Sample Characteristics

The study included 415 men referred to RL between January 1 2015 and September 31 2020. The median age at cohort entry was 38 years (interquartile range [IQR] 33–44), and almost half was Aboriginal or Torres Strait Islander (46%; Table 1). Forty-one percent of those referred to RL attended, and amongst those who did not attend, the most commonly reported reasons for not attending were no available beds, coded as no vacancy (77%), and alternative accommodation or service secured (16%; Appendix A). Those who attended RL (*n* = 170; 41%) were slightly older than those who did not attend (*n* = 245, 59%; 39 vs 38 years; *p* = 0.026) and more likely to be identified as Aboriginal or Torres Strait Islander (52% vs 41%; *p* = 0.025; Table 1). In 5 years prior to the index incarceration, participants had a median of 4 (2-7) incarcerations, 17 (IQR 8–30) charges, 5 (IQR 2–9) ED presentations and 4 (IQR 2–8) ambulance attendances. Those who attended and did not attend RL did not differ on these prior health and criminal justice events (Table 1).

**Table 1:** Demographic characteristics and health and criminal justice experiences of men referred to Rainbow Lodge January 1 2015 to September 31 2020

	Total cohort *N* = 415	Attended RL *N* = 170	Did not attend RL *N* = 245	*p*
Demographic characteristics				
Median age (years; IQR)^a^	38 (33–44)	39 (34–45)	38 (32–43)	0.026
*n* (%) Aboriginal or Torres Strait Islander status				
Yes	190 (46)	89 (52)	101 (41)	0.025
No	255 (54)	81 (48)	144 (59)	
* n* (%) Cohort entry year				
2015–2016	135 (33)	75 (44)	60 (25)	< 0.0001
2017–2018	150 (36)	56 (33)	94 (38)	
2019–2020	130 (31)	39 (23)	91 (37)	
Custodial and health contact in 5 years prior to index incarceration				
Median prior incarcerations (IQR)	4 (2–7)	5 (2–8)	4 (2–7)	0.791
Median prior days incarcerated (IQR)	888 (535–1231)	899 (546–1211)	865 (500–1231)	0.936
Median prior charges (IQR)	17 (8–30)	17 (8–29)	18 (9–31)	0.427
Median prior ED presentations (IQR)	5 (2–9)	5 (2–9)	5 (2–10)	0.788
Median prior ambulance attendance (IQR)	4 (2–8)	4 (2–8)	4 (2–9)	0.627

*RL*, rainbow lodge; *IQR*, interquartile range; *ED*, emergency department

^a^At time of release from index incarceration

#### Criminal Justice and Health Events During Follow-up Period

There was a median of 180-day follow-up per participant (IQR = 69–417) after release from index incarceration. Seventy-four percent of the sample returned to prison within the follow-up period after their index incarceration, although only 22% had a new criminal charge (*n* = 227 events) within the follow-up period (Table 2).

**Table 2:** Criminal justice and health events in the 2 years following index incarceration

	Total cohort *n* (%) *n* = 415	Attended RL *n* = 170	Did not attend RL *n* = 245	*p*
Reincarcerated				
Returned to prison within the follow-up period	295 (74)	124 (78)	171 (72)	0.17
Charges				
Charged following index incarceration	92 (22)	35 (21)	57 (23)	0.52
Median charges following index incarceration (IQR)	2 (1–3)	1 (1–3)	2 (1–3)	0.29
Emergency presentations				
Presented to ED following index incarceration	222 (53)	82 (48)	140 (57)	0.07
Median ED presentations following index incarceration (IQR)	2 (1–4)	2 (1–4)	2 (1–4)	0.03
*Triage category*				
Urgent	181 (44)	73 (43)	108 (44)	0.82
Median urgent ED presentations following index incarceration (IQR)	1 (1–2)	2 (1–3)	1 (1–2)	0.15
Low acuity	122 (29)	43 (25)	79 (32)	0.13
Median low-acuity ED presentations following index incarceration (IQR)	2 (1–3)	2 (1–3)	1 (1–2)	0.12
Ambulance attendance				
Ambulance attendance following index incarceration	160 (39)	64 (38)	96 (23)	0.75
Median ambulance attendance following index incarceration (IQR)	1 (1–2)	2 (1–2)	1 (1–3)	0.83
Deaths	6 (1)	-	-	0.65

*RL*, rainbow lodge; *IQR*, interquartile range; *ED*, emergency department; –: not reported due to small cell size

Half of the sample (53%) had at least one ED presentation (*n* = 723 events), with 44% of the total sample having an urgent ED presentation (*n* = 406) and 29% having a low-acuity presentation (*n* = 312). Five cases were not assigned a triage category. Thirty-nine percent of the sample had at least one ambulance attendance (*n* = 336), and six individuals died during the follow-up period. There was no significant difference in the sample proportion or the number of events between those who attended and those who did not attend RL on these outcomes.

#### Rates of Charges and Health Service Contact

**Fig. 1:**
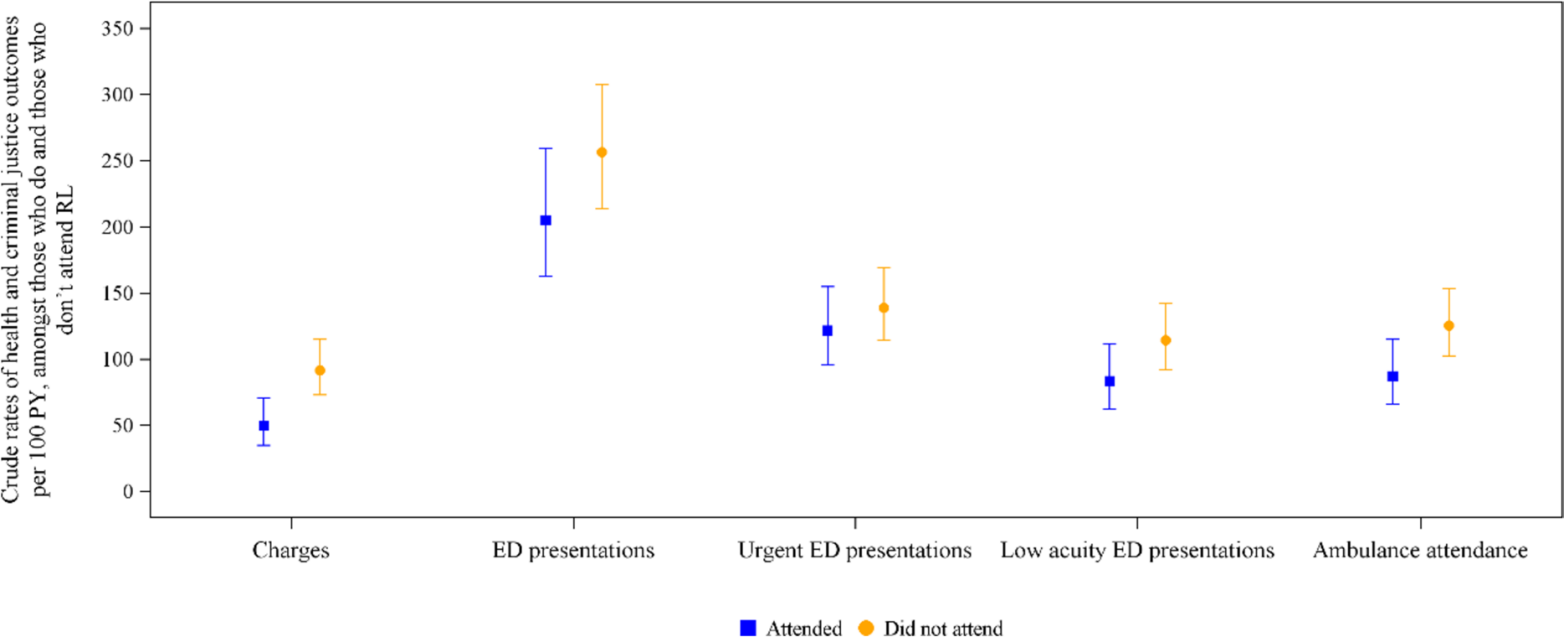
Crude rates per 100 PY of criminal charges; overall, urgent and low-acuity ED presentations; and ambulance attendance amongst those who did and did not attend RL. Figure note. PY, person-years; ED, emergency department; RL, Rainbow Lodge

Figure 1 displays the crude incidence rates per 100 PY for new criminal charges, ED presentations (total, urgent and low acuity) and ambulance attendance by RL attendance. All point estimates for rates were lower in the attender group than in those who did not attend RL.

Age, release year and Aboriginal or Torres Strait Islander status differed between the two groups and were therefore included in the multivariable model. We found strong evidence that attending RL was associated with a reduction in new criminal charges during follow-up (ARR = 0.56; 95% CI 0.340.86; *p* = 0.009). Absolute rates of ED presentations (including urgent and low acuity) and ambulance attendance were lower amongst those who attended RL than those who did not. However, there was no evidence of an association in adjusted analyses for urgent and low-acuity ED presentations (ARR = 0.88; 95%CI = 0.65–1.21), and ambulance attendance (ARR = 0.82; 95%CI = 0.57–1.18) (Table 3). The crude incidence rates, crude rate ratios and ARRs by age group, cohort entry year and prior health and criminal justice contact are presented in the supplementary material (Appendices D-H).

**Table 3:** Crude rates and adjusted rate ratios for criminal charges; overall, urgent and low-acuity ED presentations; and ambulance attendance amongst those who did and did not attend RL

	Total person-years (PY)	Total events	Crude rate per 100 PY	Adjusted rate ratio	95% CI
Charges					
Attended RL	132.95	66	49.64	0.56^a^	0.34–0.86
Not attended RL	175.54	161	91.72	Ref	Ref
ED presentations					
Attended RL	132.95	273	205.34	0.88^b^	0.65–1.21
Not attended RL	175.54	450	256.35	Ref	Ref
Urgent ED presentations					
Attended RL	132.95	162	121.85	0.99^b^	0.72–1.38
Not attended RL	175.54	244	139.00	Ref	Ref
Low-acuity ED presentations					
Attended RL	132.95	111	83.49	0.77^b^	0.53–1.14
Not attended RL	175.54	201	114.50	Ref	Ref
Ambulance attendance					
Attended RL	132.95	116	87.25	0.82^b^	0.57–1.18
Not attended RL	175.54	220	125.33	Ref	Ref

*RL*, rainbow lodge; *CI*, confidence intervals; *ED*, emergency department

^a^Adjusted for all baseline characteristics, prior number of incarcerations and prior number of charges

^b^Adjusted for all baseline characteristics, prior number of incarcerations, prior number of charges, prior ambulance attendance and prior number of ED presentations

#### Time to First Outcome Event

**Fig. 2:**
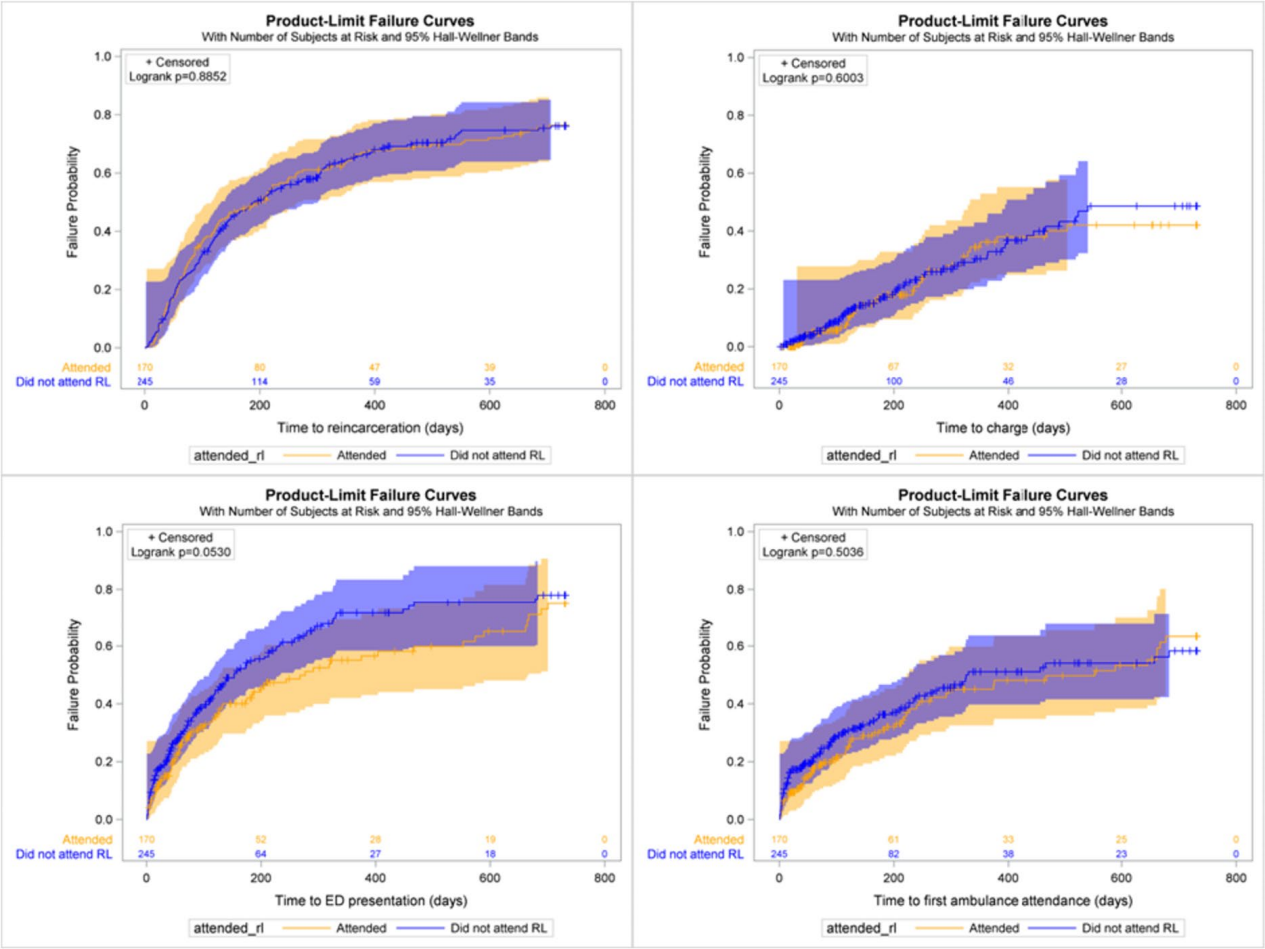
Time to first reincarceration, charge, ED presentation and ambulance attendance for RL attendees and non-attendees. Figure note. ED, emergency department; RL, Rainbow Lodge

After adjusting for potential confounding variables in Cox regression, we found no evidence of an association between attending RL and the time to first reincarceration, new criminal charge, ED presentation or ambulance attendance (Fig. 2, Table 4).

**Table 4:** Time from release from index incarceration to first ED presentation, ambulance attendance, new criminal charge and reincarceration

	Reincarceration		New criminal charges		ED presentation		Ambulance attendance	
	AHR	95% CI	AHR	95% CI	AHR	95% CI	AHR	95% CI
RL attendance								
Attended	0.98	0.76–1.25	1.06	0.68–1.66	0.82	0.60–1.12	0.84	0.58–1.22
Not attended	Ref	Ref	Ref	Ref	Ref	Ref	Ref	Ref
Age group								
< 40 years	Ref	Ref	Ref	Ref	Ref	Ref	Ref	Ref
40+ years	0.74	0.59–0.9	1.15	0.76–1.75	1.12	0.84–1.49	1.37	0.97–1.92
Release year								
2015–2016	1.66	1.2–2.29	0.56	0.31–1.02	0.96	0.65–1.42	0.75	0.47–1.19
2017–2018	1.29	1.0–1.74	0.81	0.50–1.31	0.90	0.63–1.28	0.81	0.54–1.22
2019–2020	Ref	Ref	Ref	Ref	Ref	Ref	Ref	Ref
Aboriginal or Torres Strait Islander								
Yes	Ref	Ref	Ref	Ref	Ref	Ref	Ref	Ref
No	0.86	0.68–1.09	1.36	0.88–2.10	1.17	0.87–1.57	1.49	1.04–2.13
Custodial and health contact in 5 years prior to index incarceration								
Prior incarcerations	1.07	1.04–1.11	1.07	1.00–1.14	1.03	0.98–1.08	1.01	0.95–1.07
Prior charges	1.00	0.99–1.01	1.00	0.99–1.02	1.00	0.99–1.01	1.00	0.99–1.02
Prior ambulance attendance					1.04	1.02–1.07	1.06	1.04–1.09
Prior ED presentations					1.00	0.98–1.01	0.99	0.97–1.01

*ED*, emergency department; *AHR*, adjusted hazard ratio; *CI*, confidence interval; *RL*, rainbow lodge

## Discussion

### Main Findings

This study demonstrates the feasibility of using linked administrative data to assess the impact of attending RL on health and criminal justice outcomes for men released from prison. Amongst eligible men referred to RL between 2015 and 2020, attending RL was associated with a reduced incidence rate of criminal charges. While the crude incidence rates of ED presentations and ambulance attendances were much lower for people attending RL, the CIs were wide and so no association was interpreted. Moreover, we found no evidence of an association between attending RL and time to first reincarceration, charge, ED presentation or ambulance attendance.

### Implications

The use of linked administrative data to examine the association between attending RL and select health and criminal justice outcomes has several benefits. First, administrative data precisely dates outcome events for both the exposure and control groups, rather than relying on participant recall when using self-report data. Second, by linking health and criminal justice datasets, this approach enables observation of the temporal relationship between events recorded in different datasets. Finally, this approach demonstrates the ability to use existing data to measure two of the predetermined outcomes from our co-designed model of care and program logic of supported accommodation without placing additional burden on staff or clients to report or collect data.

Despite the overall robustness and advantages of this approach, the inconsistency between the proportion of individuals who received a new charge and who were reimprisoned highlights some limitations. Although these limitations do not affect our interpretation of the strong evidence of an association between attending RL and reduced charges, they highlight the need for examining in greater detail the reason for a reimprisonment event. While the data analysed herein have utility, it is essential to consider them alongside other outcome measures, most notably those identified as relevant by RL clients and staff in the proposed co-designed best-evidence program logic for supported accommodation [19].

The rate of new criminal charges amongst those who attended RL was approximately half that amongst those who did not, although there did not appear to be a difference in time to the first new criminal charge after release, nor to first reincarceration. Existing evidence indicates that people who are conditionally released from prison (on parole) are significantly less likely to re-offend than those who are unconditionally released and that frequent contact with community corrections officers on release further reduces recidivism in the case of parole with a rehabilitative focus [30, 31]. It may be the case that the case management provided by RL enhances the effect of parole supervision demonstrated in previous research. In qualitative interviews, RL clients described challenges related to unmet practical needs and missed life skills during the post-release phase and how the structural support and direction generated at RL helped overcome them [32]. Exploring the nature and severity of charges within the two groups may increase our understanding of the impact of attending RL on criminal justice outcomes.

We found no evidence of an association between attending RL and reincarceration, although this should be interpreted with caution. Custody data used in this analysis does not detail the reason for incarceration; the inconsistency between the proportion of those charged and those reincarcerated means that reincarceration may be due to a technical violation of parole conditions, that would not result in a charge, or a charge which precedes release from index incarceration, which would further complicate efforts to understand the influence of supported accommodation on reincarceration outcomes. Like other forms of support for people in contact with the criminal justice system [33], supported accommodation has the potential to increase identification of technical violations of probation or parole conditions through more intense supervision. Detail about the reason for incarceration, including the date of the charge or if technical breaches are the cause, would increase the utility of these data to inform our understanding of the impact of supported accommodation on reincarceration.

There were substantially fewer ED visits among those who attended RL but, after adjusting for potential confounders, there was no evidence of an association between attending RL and presenting to ED, including urgent or low-acuity triage categories. While ED is intended for acute or urgent health issues, it is a known pathway to hospital, that all men (those who did and those who did not attend RL) may use to address health issues which went unchecked while in prison. Therefore, ED access could be an indication of urgent health harms, or uptake of ED services in the absence of access to primary health services.

Greater detail regarding the nature of emergency health service, including ED and ambulance, contact may better align these measures with outcomes that reflect the intended impact of supported accommodation for people released from prison (e.g. quality of life and self-efficacy) [19]. More frequent emergency health service contact is associated with increased likelihood of reincarceration [24], and use of these services following release from prison is commonly related to alcohol, other drugs or mental health [22, 23, 34]. The relationship between these health issues and (re)incarceration has previously been demonstrated [35–38], and this increase may be an indication of poor personal well-being or quality of life. Greater granularity of information about reason for presentation and treatment upon contact with emergency health service will enhance the use of these data in assessing the impact of attending RL on relevant outcomes.

### Strengths and Limitations

While this study demonstrated the feasibility of using linked administrative data to assess the impact of attending RL on criminal charges, we were unable to assess other outcomes related to supported accommodation services, such as quality of life and self-efficacy. A complete evaluation of the impact of attending RL would necessarily include appropriate measures of all outcomes of interest (including self-efficacy and quality of life). Linked administrative data are primarily intended for clinical care and administration, not research. The data used in this study do not include information about the duration of attendance at RL; start dates were estimated based on prison release dates, but we lack exit dates, therefore cannot be certain that all men in the RL attendee group were engaged with RL for the measured exposure time (i.e. 12 weeks). Data on the housing or support experiences of those who did not attend RL are also incomplete, and it is unknown whether men received comparable support elsewhere or were housed through other means. As a result, this may have biased our findings towards the null. The small size of our sample may also have contributed to null findings. However, by pooling referrals to RL between January 1 2015 and September 31 2020, our study is likely to have provided the largest sample size possible without compromising the relevance of our findings to the current service and criminal justice context in NSW. Finally, because these data are retrospective and do not include details of RL participation (meaning activities or program components), this study is limited by the inability to assess the impact of specific program elements.

Integrating linked administrative data into future evaluations that also include process measures to assess program implementation will provide a fuller understanding of whether supported accommodation achieves its intended outcomes. The high proportion of those who did not attend RL due to no vacancy highlights the high demand for these services and consequently the need for rigorous evidence to support their delivery.

## Conclusion

Our findings corroborate other evidence that suggests housing and incarceration have a strong link, because attending RL was associated with a reduced rate of re-offending. While there were weak relationships between attending RL and other outcome measures, the period following release from prison is complex and demanding, and supported accommodation is only one component of the support required to overcome the challenges men experience during this time. Integrating linked administrative data with measures of impact on quality of life and self-efficacy may paint a more complete picture of how supported accommodation is meeting its intended aims.

### Supplementary Information

ESM 1
