# Supplementary material for: Associations Between Supported Accommodation and Health and Re-offending Outcomes: a Retrospective Data Linkage Study
Source: J Urban Health. 2024 Feb 13;101(1):80–91. doi: 10.1007/s11524-023-00824-w (PMC10897077; doi:10.1007/s11524-023-00824-w)
Supplement: Supplementary file 1 — ESM 1 [file 11524_2023_824_MOESM1_ESM.docx]

**Supplementary material: Associations between supported accommodation and health and reoffending outcomes: A retrospective data linkage study**

[Appendix A: RECORD statement checklist items 2](#_Toc143695050)

[Appendix B: Reasons for not attending Rainbow Lodge 5](#_Toc143695051)

[Appendix C: Flow chart of data linkage and included participants. 6](#_Toc143695052)

[Appendix D: Crude incidence rate and unadjusted and adjusted rate ratios of new criminal charges by Rainbow Lodge attendance status, year, demographic, prior health and criminal justice characteristics. 7](#_Toc143695053)

[Appendix E: Crude incidence rate and unadjusted and adjusted rate ratios of all emergency department presentations by Rainbow Lodge attendance status, year, demographic, prior health and criminal justice characteristics. 8](#_Toc143695054)

[Appendix F: Crude incidence rate and unadjusted and adjusted rate ratios of urgent emergency department presentations by Rainbow Lodge attendance status, year, demographic, prior health and criminal justice characteristics. 9](#_Toc143695055)

[Appendix G: Crude incidence rate and unadjusted and adjusted rate ratios of low acuity emergency department presentations by Rainbow Lodge attendance status, year, demographic, prior health and criminal justice characteristics. 10](#_Toc143695056)

[Appendix H: Crude incidence rate and unadjusted and adjusted rate ratios of ambulance attendance by Rainbow Lodge attendance status, year, demographic, prior health and criminal justice characteristics. 11](#_Toc143695057)

Appendix A: RECORD statement checklist items

|  | **Item No.** | **RECORD items** | **Location in manuscript where items are reported** |
| --- | --- | --- | --- |
| **Title and abstract** | | | |
|  | 1 | RECORD 1.1: The type of data used should be specified in the title or abstract. When possible, the name of the databases used should be included.  RECORD 1.2: If applicable, the geographic region and timeframe within which the study took place should be reported in the title or abstract.  RECORD 1.3: If linkage between databases was conducted for the study, this should be clearly stated in the title or abstract. | 1-2 |
| **Introduction** | | | |
| Background rationale | 2 | Explain the scientific background and rationale for the investigation being reported | 3-4 |
| Objectives | 3 | State specific objectives, including any prespecified hypotheses | 4 |
| **Methods** | | | |
| Study Design | 4 | Present key elements of study design early in the paper | 4 |
| Setting | 5 | Describe the setting, locations, and relevant dates, including periods of recruitment, exposure, follow-up, and data collection | 4-5 |
| Participants | 6 | RECORD 6.1: The methods of study population selection (such as codes or algorithms used to identify subjects) should be listed in detail. If this is not possible, an explanation should be provided.  RECORD 6.2: Any validation studies of the codes or algorithms used to select the population should be referenced. If validation was conducted for this study and not published elsewhere, detailed methods and results should be provided.  RECORD 6.3: If the study involved linkage of databases, consider use of a flow diagram or other graphical display to demonstrate the data linkage process, including the number of individuals with linked data at each stage. | 4-5, Appendix B |
| Variables | 7 | RECORD 7.1: A complete list of codes and algorithms used to classify exposures, outcomes, confounders, and effect modifiers should be provided. If these cannot be reported, an explanation should be provided. | 6-7 |
| Data sources/ measurement | 8 | For each variable of interest, give sources of data and details of methods of assessment (measurement).  Describe comparability of assessment methods if there is more than one group | 6-7 |
| Bias | 9 | Describe any efforts to address potential sources of bias |  |
| Study size | 10 | Explain how the study size was arrived at | 4-5, Appendix B |
| Quantitative variables | 11 | Explain how quantitative variables were handled in the analyses. If applicable, describe which groupings were chosen, and why |  |
| Statistical methods | 12 | (a) Describe all statistical methods, including those used to control for confounding  (b) Describe any methods used to examine subgroups and interactions  (c) Explain how missing data were addressed  (d) *Cohort study* - If applicable, explain how loss to follow-up was addressed  *Case-control study* - If applicable, explain how matching of cases and controls was addressed  *Cross-sectional study* - If applicable, describe analytical methods taking account of sampling strategy  (e) Describe any sensitivity analyses |  |
| Data access and cleaning methods |  | RECORD 12.1: Authors should describe the extent to which the investigators had access to the database population used to create the study population.  RECORD 12.2: Authors should provide information on the data cleaning methods used in the study. |  |
| Linkage |  | RECORD 12.3: State whether the study included person-level, institutional-level, or other data linkage across two or more databases. The methods of linkage and methods of linkage quality evaluation should be provided. | 6 |
| **Results** | | | |
| Participants | 13 | RECORD 13.1: Describe in detail the selection of the persons included in the study (*i.e.,* study population selection) including filtering based on data quality, data availability and linkage. The selection of included persons can be described in the text and/or by means of the study flow diagram. | 4-5, Appendix B |
| Descriptive data | 14 | (a) Give characteristics of study participants (*e.g.*, demographic, clinical, social) and information on exposures and potential confounders  (b) Indicate the number of participants with missing data for each variable of interest  (c) *Cohort study* - summarise follow-up time (*e.g.*, average and total amount) |  |
| Outcome data | 15 | *Cohort study* - Report numbers of outcome events or summary measures over time  *Case-control study* - Report numbers in each exposure category, or summary measures of exposure  *Cross-sectional study* - Report numbers of outcome events or summary measures |  |
| Main results | 16 | (a) Give unadjusted estimates and, if applicable, confounder-adjusted estimates and their precision (e.g., 95% confidence interval). Make clear which confounders were adjusted for and why they were included  (b) Report category boundaries when continuous variables were categorized  (c) If relevant, consider translating estimates of relative risk into absolute risk for a meaningful time period |  |
| Other analyses | 17 | Report other analyses done—e.g., analyses of subgroups and interactions, and sensitivity analyses |  |
| **Discussion** | | | |
| Key results | 18 |  |  |
| Limitations | 19 | RECORD 19.1: Discuss the implications of using data that were not created or collected to answer the specific research question(s). Include discussion of misclassification bias, unmeasured confounding, missing data, and changing eligibility over time, as they pertain to the study being reported. |  |
| Interpretation | 20 | Give a cautious overall interpretation of results considering objectives, limitations, multiplicity of analyses, results from similar studies, and other relevant evidence |  |
| Generalisability | 21 | Discuss the generalisability (external validity) of the study results |  |
| **Other Information** | | | |
| Funding | 22 | Give the source of funding and the role of the funders for the present study and, if applicable, for the original study on which the present article is based |  |
| Accessibility of protocol, raw data, and programming code |  | RECORD 22.1: Authors should provide information on how to access any supplemental information such as the study protocol, raw data, or programming code. |  |

*Reference: Benchimol EI, Smeeth L, Guttmann A, Harron K, Moher D, Petersen I, Sørensen HT, von Elm E, Langan SM, the RECORD Working Committee.  The REporting of studies Conducted using Observational Routinely-collected health Data (RECORD) Statement.  *PLoS Medicine* 2015; in press.

*Checklist is protected under Creative Commons Attribution ([CC BY](http://creativecommons.org/licenses/by/4.0/)) license.

Appendix B: Reasons for not attending Rainbow Lodge

| Reasons for not attending | N | % |
| --- | --- | --- |
| Already released from prison | - | - |
| Alternative service/accommodation secured | 63 | 16 |
| No contact following release | 6 | 2 |
| No vacancy | 295 | 77 |
| Not interested in attending RL | 19 | 5 |
| **Total** | **385** | **100** |

**Table note**: –: Not reported due to small cell size.

Appendix C: Flow chart of data linkage and included participants.


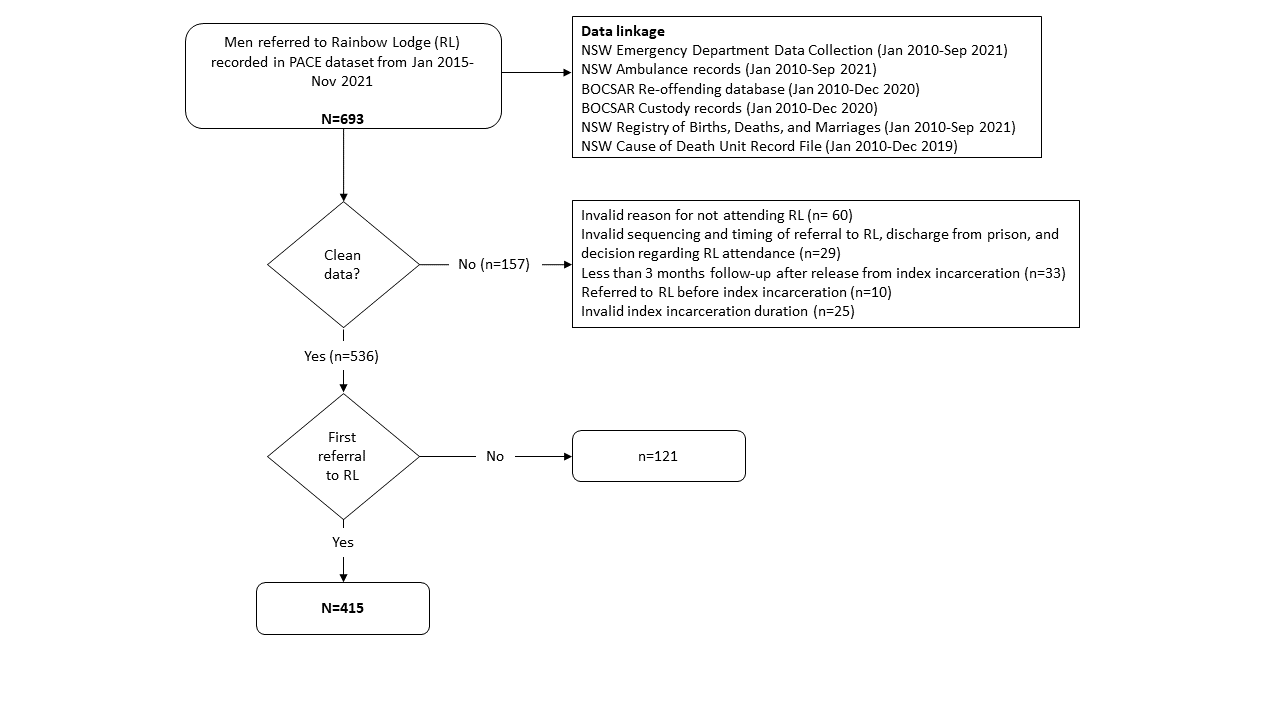


Appendix D: Crude incidence rate and unadjusted and adjusted rate ratios of new criminal charges by Rainbow Lodge attendance status, year, demographic, prior health and criminal justice characteristics.

|  | **Count** | **Person-years (PY)** | **RL crude rate per 100 PY** | **Crude rate ratio** | **95% Confidence interval** | **Adjusted rate ratio** | **95% Confidence interval** |
| --- | --- | --- | --- | --- | --- | --- | --- |
| Overall | 227 | 308.49 |  |  |  |  |  |
| **RL Status** |  |  |  |  |  |  |  |
| Attended RL | 66 | 132.95 | 49.64 | 0.54 | 0.37-0.82 | 0.56 | 0.37-0.87 |
| Not attended RL | 161 | 175.54 | 91.72 | ref | ref | ref | ref |
| **Age group** |  |  |  |  |  |  |  |
| <40 years | 98 | 157.24 | 62.32 | ref | ref | ref | ref |
| ≥40 years | 129 | 151.24 | 66.12 | 1.37 | 0.93-2.02 | 1.42 | 0.97-2.09 |
| **Indigenous status** |  |  |  |  |  |  |  |
| Non-Indigenous | 163 | 180.37 | 90.37 | 1.18 | 1.18-2.76 | 1.99 | 1.29-3.07 |
| Indigenous | 64 | 128.12 | 49.95 | ref | ref | ref | ref |
| **Cohort entry year** |  |  |  |  |  |  |  |
| 2015-2016 | 63 | 102.93 | 61.20 | 0.75 | 0.45-1.24 | 0.90 | 0.53-1.52 |
| 2017-2018 | 97 | 123.94 | 78.26 | 0.95 | 0.60-1.51 | 1.10 | 0.70-1.73 |
| 2019-2020 | 67 | 81.61 | 82.10 | ref | ref | ref | ref |
| **Custodial and health contact in the 5 years prior to index incarceration** |  |  |  |  |  |  |  |
| Prior incarcerations |  |  |  | 1.82 | 1.23-2.70 | 0.66 | 0.86-1.21 |
| Prior charges |  |  |  | 1.13 | 1.01-1.27 | 1.93 | 1.05-3.53 |
| Prior ambulance attendance |  |  |  | 1.15 | 0.96-1.38 |  |  |
| Prior ED presentations |  |  |  | 1.12 | 0.98-1.27 |  |  |

Appendix E: Crude incidence rate and unadjusted and adjusted rate ratios of all emergency department presentations by Rainbow Lodge attendance status, year, demographic, prior health and criminal justice characteristics.

|  | **Count** | **Person-years (PY)** | **RL crude rate per 100 PY** | **Crude rate ratio** | **95% Confidence interval** | **Adjusted rate ratio** | **95% Confidence interval** |
| --- | --- | --- | --- | --- | --- | --- | --- |
| Overall | 723 | 308.49 |  |  |  |  |  |
| **RL Status** |  |  |  |  |  |  |  |
| Attended RL | 273 | 132.95 | 205.34 | 0.80 | 0.60-1.08 | 0.88 | 0.65-1.21 |
| Not attended RL | 450 | 175.54 | 256.36 | ref | ref | ref | ref |
| **Age group** |  |  |  |  |  |  |  |
| <40 years | 367 | 157.24 | 233.40 | ref | ref | ref | ref |
| ≥40 years | 356 | 151.24 | 235.38 | 1.01 | 0.76-1.34 | 1.03 | 0.77-1.38 |
| **Indigenous status** |  |  |  |  |  |  |  |
| Non-Indigenous | 418 | 180.37 | 231.75 | 0.97 | 0.73-1.30 | 0.89 | 0.65-1.21 |
| Indigenous | 305 | 128.12 | 238.06 | ref | ref | ref | ref |
| **Cohort entry year** |  |  |  |  |  |  |  |
| 2015-2016 | 187 | 102.93 | 181.67 | 0.63 | 0.43-0.91 | 0.65 | 0.44-0.98 |
| 2017-2018 | 299 | 123.94 | 241.25 | 0.83 | 0.60-1.16 | 0.85 | 0.61-1.19 |
| 2019-2020 | 237 | 81.61 | 290.40 | ref | ref | ref | ref |
| **Custodial and health contact in the 5 years prior to index incarceration** |  |  |  |  |  |  |  |
| Prior incarcerations |  |  |  | 2.34 | 1.85-2.96 | 1.38 | 0.56-2.21 |
| Prior charges |  |  |  | 1.20 | 1.11-1.30 | 1.01 | 0.89-1.14 |
| Prior ambulance attendance |  |  |  | 1.54 | 1.44-1.66 | 1.62 | 1.36-1.93 |
| Prior ED presentations |  |  |  | 1.34 | 1.26-1.42 | 0.92 | 0.80-1.07 |

Appendix F: Crude incidence rate and unadjusted and adjusted rate ratios of urgent emergency department presentations by Rainbow Lodge attendance status, year, demographic, prior health and criminal justice characteristics.

|  | **Count** | **Person-years (PY)** | **RL crude rate per 100 PY** | **Crude rate ratio** | **95% Confidence interval** | **Adjusted rate ratio** | **95% Confidence interval** |
| --- | --- | --- | --- | --- | --- | --- | --- |
| Overall | 406 | 308.49 |  |  |  |  |  |
| **RL Status** |  |  |  |  |  |  |  |
| Attended RL | 162 | 132.95 | 121.85 | 0.88 | 0.64-1.20 | 0.99 | 0.72-1.38 |
| Not attended RL | 244 | 175.54 | 139.00 | ref | ref | ref | ref |
| **Age group** |  |  |  |  |  |  |  |
| <40 years | 202 | 157.24 | 128.46 | ref | ref | ref | ref |
| ≥40 years | 204 | 151.24 | 134.88 | 1.05 | 0.78-1.42 | 1.05 | 0.77-1.43 |
| **Indigenous status** |  |  |  |  |  |  |  |
| Non-Indigenous | 240 | 180.37 | 133.06 | 1.03 | 0.75-1.40 | 1.09 | 0.79-1.49 |
| Indigenous | 166 | 128.12 | 129.57 | ref | ref | ref | ref |
| **Cohort entry year** |  |  |  |  |  |  |  |
| 2015-2016 | 10 | 102.93 | 100.06 | 0.59 | 0.40-0.88 | 0.58 | 0.38-0.89 |
| 2017-2018 | 165 | 123.94 | 133.13 | 0.79 | 0.55-1.12 | 0.79 | 0.56-1.13 |
| 2019-2020 | 138 | 81.61 | 169.09 | ref | ref | ref | ref |
| **Custodial and health contact in the 5 years prior to index incarceration** |  |  |  |  |  |  |  |
| Prior incarcerations |  |  |  | 2.28 | 1.77-2.95 | 1.21 | 0.73-2.01 |
| Prior charges |  |  |  | 1.21 | 1.12-1.31 | 1.02 | 0.90-1.15 |
| Prior ambulance attendance |  |  |  | 1.59 | 1.49-1.71 | 2.00 | 1.69-2.37 |
| Prior ED presentations |  |  |  | 1.34 | 1.26-1.43 | 0.81 | 0.70-0.94 |

Appendix G: Crude incidence rate and unadjusted and adjusted rate ratios of low acuity emergency department presentations by Rainbow Lodge attendance status, year, demographic, prior health and criminal justice characteristics.

|  | **Count** | **Person-years (PY)** | **RL crude rate per 100 PY** | **Crude rate ratio** | **95% Confidence interval** | **Adjusted rate ratio** | **95% Confidence interval** |
| --- | --- | --- | --- | --- | --- | --- | --- |
| Overall | 312 | 308.49 |  |  |  |  |  |
| **RL Status** |  |  |  |  |  |  |  |
| Attended RL | 111 | 132.95 | 62.49 | 0.73 | 0.51-1.05 | 0.77 | 0.53-1.14 |
| Not attended RL | 201 | 175.54 | 92.32 | ref | ref | ref | ref |
| **Age group** |  |  |  |  |  |  |  |
| <40 years | 161 | 157.24 | 102.39 | ref | ref | ref | ref |
| ≥40 years | 151 | 151.24 | 99.84 | 0.98 | 0.69-1.38 | 1.02 | 0.72-1.45 |
| **Indigenous status** |  |  |  |  |  |  |  |
| Non-Indigenous | 174 | 180.37 | 76.48 | 0.90 | 0.63-1.27 | 0.92 | 0.64-1.61 |
| Indigenous | 138 | 128.12 | 82.99 | ref | ref | ref | ref |
| **Cohort entry year** |  |  |  |  |  |  |  |
| 2015-2016 | 84 | 102.93 | 81.60 | 0.68 | 0.43-1.07 | 0.76 | 0.47-1.23 |
| 2017-2018 | 130 | 123.94 | 104.89 | 0.87 | 0.58-1.32 | 0.90 | 0.60-1.37 |
| 2019-2020 | 98 | 81.61 | 120.08 | ref | ref | ref | ref |
| **Custodial and health contact in the 5 years prior to index incarceration** |  |  |  |  |  |  |  |
| Prior incarcerations |  |  |  | 2.44 | 1.85-3.21 | 1.56 | 0.87-2.81 |
| Prior charges |  |  |  | 1.20 | 1.09-1.32 | 1.00 | 0.85-1.18 |
| Prior ambulance attendance |  |  |  | 1.48 | 1.34-1.63 | 1.20 | 0.94-1.54 |
| Prior ED presentations |  |  |  | 1.34 | 1.24-1.44 | 1.11 | 0.92-1.34 |

Appendix H: Crude incidence rate and unadjusted and adjusted rate ratios of ambulance attendance by Rainbow Lodge attendance status, year, demographic, prior health and criminal justice characteristics.

|  | **Count** | **Person-years (PY)** | **RL crude rate per 100 PY** | **Crude rate ratio** | **95% Confidence interval** | **Adjusted rate ratio** | **95% Confidence interval** |
| --- | --- | --- | --- | --- | --- | --- | --- |
| Overall | 336 | 308.49 |  |  |  |  |  |
| **RL Status** |  |  |  |  |  |  |  |
| Attended RL | 116 | 132.95 | 87.25 | 0.70 | 0.50-0.98 | 0.82 | 0.57-1.18 |
| Not attended RL | 220 | 175.54 | 125.33 | ref | ref | ref | ref |
| **Age group** |  |  |  |  |  |  |  |
| <40 years | 170 | 157.24 | 105.57 | ref | ref | ref | ref |
| ≥40 years | 166 | 151.24 | 112.40 | 1.06 | 0.77-1.48 | 1.08 | 0.78-1.50 |
| **Indigenous status** |  |  |  |  |  |  |  |
| Non-Indigenous | 208 | 180.37 | 115.32 | 1.15 | 0.83-1.61 | 0.20 | 0.86-1.69 |
| Indigenous | 128 | 128.12 | 99.91 | ref | ref | ref | ref |
| **Cohort entry year** |  |  |  |  |  |  |  |
| 2015-2016 | 80 | 102.93 | 77.72 | 0.48 | 0.32-0.72 | 0.50 | 0.32-0.77 |
| 2017-2018 | 123 | 123.94 | 99.24 | 0.61 | 0.42-0.88 | 0.64 | 0.44-0.92 |
| 2019-2020 | 133 | 81.61 | 162.26 | ref | ref | ref | ref |
| **Custodial and health contact in the 5 years prior to index incarceration** |  |  |  |  |  |  |  |
| Prior incarcerations |  |  |  | 2.09 | 1.56-2.82 | 1.04 | 0.59-1.85 |
| Prior charges |  |  |  | 1.19 | 1.09-1.30 | 1.03 | 0.90-1.17 |
| Prior ambulance attendance |  |  |  | 1.58 | 1.46-1.71 | 1.93 | 1.60-2.62 |
| Prior ED presentations |  |  |  | 1.35 | 1.26-1.44 | 0.84 | 0.72-0.99 |
